# Supplementary material for: Coping Resources among Forced Migrants in South Africa: Exploring the Role of Character Strengths in Coping, Adjustment, and Flourishing
Source: Int J Environ Res Public Health. 2023 Dec 29;21(1):50. doi: 10.3390/ijerph21010050 (PMC10815753; doi:10.3390/ijerph21010050)
Supplement: Supplementary file 1 [file ijerph-21-00050-s001.zip › ijerph-2563552-Supplementary Material.pdf]

Supplementary Table S1. Themes of Character Strength in African Asylees' and Refugees' Narratives (*N* = 14)

Theme	Percent	Example Quotes
Spirituality and Religiousness	64.3	"The Spirit is leading us, even where we are today, we are still strong, that is how we are living."<br>"I am praying a lot. I am not dreaming about it …prayer helped me to forget the traumatic experience."<br>"Going to church helped me to recover slowly…the support from the pastor and the church members."<br>"I believe that peoples' situation is determined by God, not by people… I have faith in God…my Christian faith and the support I get from the church and the pastor keeps me together. I am encouraged by the Gospel and the Bible."<br>"I am still alive because of God. God is the reason that I am still alive. Everything from God."<br>"I keep God in my heart, it is helping, things will be right… I find some ways to deal [with challenges]. God has His ways."<br>"I was praying to arrive safely, and I was hopeful that by God's wishes, my family was safe."
Kindness and Love	57.1	"When I look at my wife and two sons, I feel so happy, and encouraged to do even more."<br>"I have kids that I am raising, and I need to stay to raise them until they grow up. That is what keeps me going."<br>"When [I] started connecting and meeting people from the same journey… I started opening up."<br>"The family of Rasta support one another, we give ideas to one another… very big support, more than money and silver."<br>"When I gave birth to my first child, I was very happy to see my child alive. It relieved my stress from the traumatic experience."<br>"My wife supports me a lot… We have aimed at something, and we are working together [toward our] objective and we will achieve it. I am seeing really… a very good life."<br>"To keep strength and my life going... the courage [support] from people... keep me out of the [negative] emotions"<br>"I have to stand and overcome challenges to accomplish the expected responsibility for my children."
Hope and Optimism	44.5	"I always think that something good to happen, so I live by hope… things will change for good."<br>"There was [a] time I felt my life was ending... but I also realized that I did not know what the future will bring."<br>"I am optimist things will change, and a new day will come and new things as well… One thing is to focus, focus and to look for opportunity."<br>"My belief that I always carry with me… no matter the situation I am in those things pass… I live hoping for better."<br>"I am always an optimist. In fact, I did another course to upgrade myself."<br>"I am building a future because I am not returning… I must be patient and never lose hope."
Persistence and Fortitude	28.6	"Although I was discouraged, I was also coming back and make myself ready to try other ways."<br>"There was such moment [I said] this is the end of my life, but there was a feeling of keep telling me keep trying keep trying… "<br>"I did not want to remain a car guard, I had to do something. Something, inside me, told me that a car guard was not who I am and what I wanted to be. When I decided to learn… My energy was coming, and I want to further my studies."
Gratitude and Thankfulness	21.4	"It is grace to come alive from that place ...now I am trying to take things easy [because] my past hardship helped me to see things easily."<br>"I am fortunate enough to run away from the country."<br>"The fact that I am alive, I thank God and that gave me the strength and I hope life will be ok."
